# Neuronal oscillations and functional connectivity of paced nostril breathing: A high-density EEG study

**DOI:** 10.1371/journal.pone.0316125

**Published:** 2025-02-03

**Authors:** Anita B. Frohlich, Flavio Frohlich, Miriam Sklerov

**Affiliations:** 1 Department of Neurology, University of North Carolina, Chapel Hill, North Carolina, United States of America; 2 Department of Psychiatry, University of North Carolina, Chapel Hill, North Carolina, United States of America; 3 Carolina Center for Neurostimulation, University of North Carolina, Chapel Hill, North Carolina, United States of America; 4 Neuroscience Center, University of North Carolina, Chapel Hill, North Carolina, United States of America; 5 Department of Cell Biology and Physiology, University of North Carolina, Chapel Hill, North Carolina, United States of America; 6 Department of Biomedical Engineering, University of North Carolina, Chapel Hill, North Carolina, United States of America; Institute of Psychology Chinese Academy of Sciences, CHINA

## Abstract

Controlling nostril airflow through hand manipulations is an ancient yoga technique that has been suggested to provide targeted modulation of neuronal excitability and regulation of autonomic function, which is known to be lateralized in the brain. Here, we examined if unilateral and alternate nostril breathing differentially impacts brain network oscillations measured by high-density EEG in healthy control participants with no prior experience in breathing techniques. We found that paced nostril breathing both decreased alpha/mu oscillations over central and parietal areas and increased frontal midline and occipital theta oscillations when comparing to spontaneous breathing. Alternate nostril breathing suppressed alpha/mu oscillation more than left nostril breathing. Unilateral nostril breathing resulted in an ipsilateral increase in alpha connectivity while left nostril breathing increased anterior-posterior midline theta connectivity. In contrast to the EEG results, heart rate, heart rate variability, and cognitive performance assessed with a working memory task did not differ significantly by breathing condition. Our results add to the existent literature on nasal breathing by demonstrating changes in cortical oscillations and connectivity during a task that combined slow breathing with manual nasal pathway modulation.

## Introduction

Nostril breathing is typically unilateral at any single time, cycling between the left and right on a timescale of minutes to hours [1]. Periodically, the erectile tissue in the anterior part of the nasal septum and the inferior turbinate of one nostril enlarge resulting in a reduction of airflow such that air predominantly flows through the contralateral nostril [2]. After 30 minutes to several hours, the airflow dominance shifts to the other nostril [3]. This nasal cycle seems to be largely driven by the autonomic nervous system [4], but the underlying physiological mechanism and functional implications are not yet understood.

Interestingly, in yoga, some breathing techniques (pranayama) intentionally attempt to alter the nasal cycle. Specifically, a specific hand shape (mudra) is used to close the left nostril, right nostril, or both nostrils alternatingly (nadi shodhana). The claim is that cognitive, physiological, and psychological states can be altered by manipulating nasal airflow. Unilaterally breathing through one nostril is thought to stimulate the contralateral hemisphere, while alternating between both nostrils is said to equilibrate brain activity [5]. Although these techniques have a long history, scientific exploration of these proposed effects and their underlying physiology has been sparse and recent.

One approach to investigate the neural correlates of nostril breathing is using electroencephalography (EEG), which provides high temporal resolution to measure synchronized brain activity that results from rhythmic coordination of neuronal populations. Different EEG frequencies have been associated with specific neuronal networks, states, and functions [6]. Synchronization of neuronal oscillations establishes long-range functional connectivity and provides a mechanism for communication within functional brain networks [7]. EEG thus represents a promising tool to investigate how nostril breathing correlates with brain states and network dynamics. Previous studies have used EEG to explore a possible link between nostril breathing and brain activity. The data from these prior studies, however, is ambivalent with regards to lateralization of brain function as a result of alternate nostril breathing [8–12]. Robust support for any of the proposed EEG mechanisms associated with nostril breathing techniques is missing. The small number of studies combined with the typically used, unique study population of people experienced in breathing techniques further limit the interpretability and generalizability of the findings. Fundamentally, it remains unclear if manually closing a nostril changes brain activity in terms of neural oscillations and functional connectivity measured by EEG.

To address this gap in the literature, we here investigate if paced breathing while manually closing the left, right, or both nostrils differentially affects brain network oscillations in people without prior practice in breathing techniques. We hypothesized that paced left, right, and alternate nostril breathing with manual manipulation of the nostrils distinctively modulates brain network oscillations associated both with sensorimotor and cognitive control. We further hypothesize that breathing through the left or right nasal pathway will be reflected in neuronal asymmetry with ipsilateral inhibition. To test these hypotheses, we used high-density EEG to measure brain activity during a single-visit study in which blocks of computer-paced nostril breathing were interleaved with a working memory task to control overall behavior state.

## Materials & methods

### Ethical approval

The procedures followed in this study were in accordance with the 2013 version of the *Declaration of Helsinki*. The study was approved by the local institutional review board (UNC-CH IRB; protocol ID: 22–1198). Participants were recruited via the Research for Me @ UNC website in September and October 2022 and provided written consent before study participation.

### Participants

After completing an online prescreen and a brief phone screening, 19 individuals were enrolled in the study between September 17^th^, 2022, and October 21^st^, 2022. Participant inclusion and exclusion criteria were as follows: (1) 18 years and older; (2) English speaker; (3) not pregnant; (4) no psychiatric or neurological disorder or condition; (5) no current seasonal allergies or respiratory symptoms; (6) no yoga, pranayama (breathing techniques), or meditation experience; (7) a BMI ≤ 30. All participants received monetary compensation for their time. Participant demographics are shown in Table 1.

**Table 1:** Participant demographics.

N = 19	N (%)
***Sex***	
Cisgender Male	7 (36.8)
Cisgender Female	12 (63.2)
***Race/Ethnicity***	
Caucasian	12 (63.2)
Black/African American	2 (10.5)
Asian	3 (15.8)
Hispanic/Latinx	2 (10.5)
***Age***	
18–24	3 (15.8)
25–34	9 (47.3)
35–44	3 (15.8)
45–54	1 (5.3)
55–64	1 (5.3)
Over 65	2 (10.5)
***Highest Level of Education***	
Some college, no degree	1 (5.3)
Bachelor’s degree	7 (36.8)
Some post-undergraduate work	3 (15.8)
Advanced degree	8 (42.1)
***Handedness***	
Predominantly right	17 (89.5)
Predominantly left	2 (10.5)

### Experimental design and procedures

**Fig 1:**
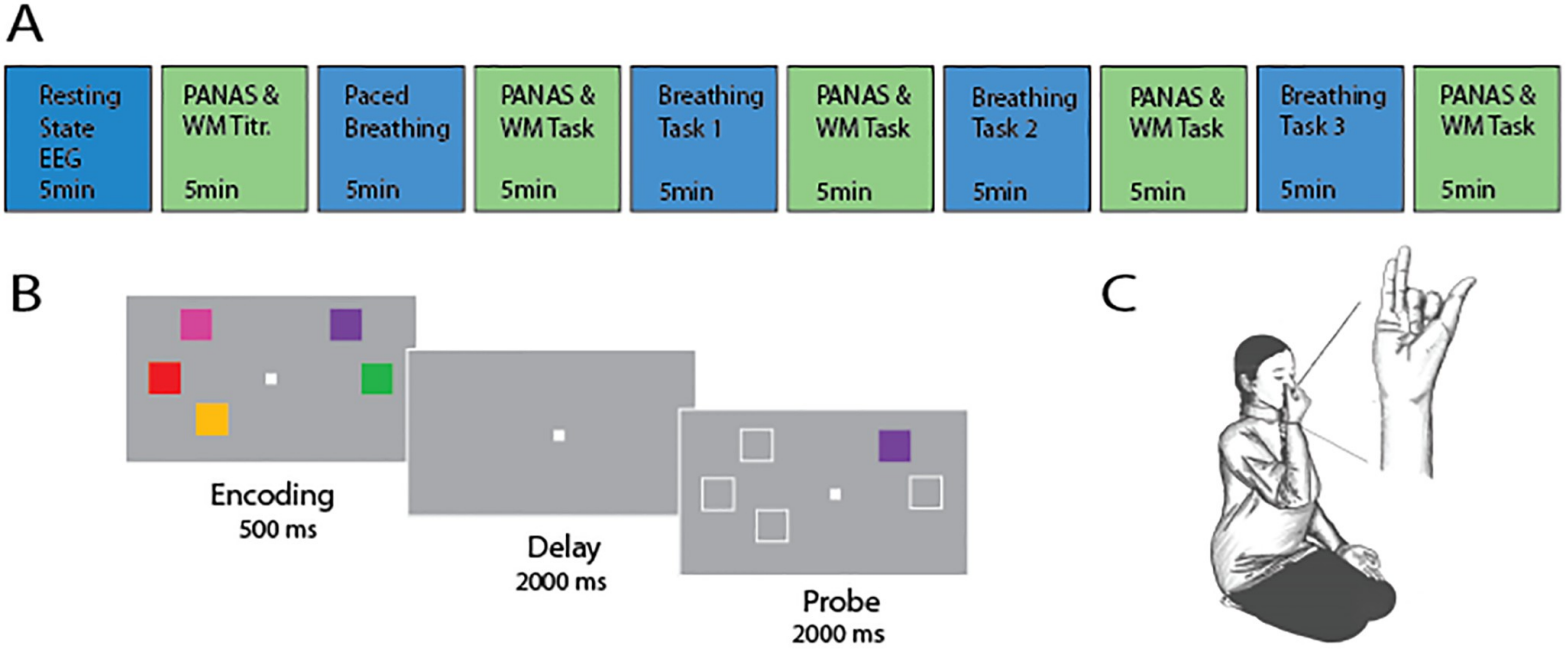
Experimental design and procedures. A: The experiment comprised ten 5-minute intervals—a resting state recording, followed by a PANAS and a working memory titration, a breathing practice instructing participants in the three nostril breathing techniques as well as computer-paced breathing, followed by a PANAS and a working memory task, then looping through the three nostril breathing conditions in randomized order, each followed by a PANAS and a working memory task. B: The working memory task consisted of three epochs—encoding, delay, and probe (illustration from [13]). C: Picture of the mudra (hand shape) which was used by participants to close nostrils to manipulate nasal airflow [14].

The experimental sequence is illustrated in Fig 1. The experiment was conducted in the Carolina Center for Neurostimulation in the Department of Psychiatry at the University of North Carolina at Chapel Hill. To maintain uniformity across the study, all sessions were consistently held in a designated environment, utilizing standardized equipment. Additionally, a single researcher provided oversight to ensure methodological consistency and minimize potential confounding variables.

#### Electroencephalography and physiologic recording

EEG data were collected with a sampling rate of 1000 Hz using a 128-channel high-density electrode net (HydroCel-128; Electrical Geodesics, Inc., Portland OR) connected to an EGI amplifier (NetAmps 410, Electrical Geodesics, Inc.). The impedance of each electrode was required to be <50kΩ at the start of each data collection. For the cardiorespiratory measurements, we used an EGI Physio16 input box (Electrical Geodesics, Inc.). Participants were fitted with a breathing belt around their abdomen to measure respiratory frequency. Electrocardiogram (ECG) was recorded with two electrodes below the right collar bone and the left lower ribcage.

#### Eyes-open and eyes-closed resting state EEG

At the beginning of the session, we recorded 3 minutes of eyes-open and 2 minutes of eyes-closed resting state EEG as a baseline measurement. During the eye-open condition, participants were instructed to keep their eyes on a central fixation cross, to relax their body, and to let their mind wander. For the resting-state EEG, participants were not yet instructed in the different breathing techniques and were told not to focus on anything specific, including their breathing.

#### Visuospatial working memory task

Participants performed a visuospatial working memory task after the resting-state baseline EEG as well as after each breathing condition. The working memory task required participants to memorize an array of colored squares like previous studies on working memory in our lab [13]. The task consisted of three epochs: encoding, delay, and probe (Fig 1). During the encoding phase, participants were shown a varying number of colored squares for 500ms and instructed to note color and location of the squares. This was followed by a delay period of 2000ms during which the squares disappeared. Participants were subsequently probed on their memory: they were shown the same arrangement of squares as in the encoding phase but only one of the squares was colored. Participants used their index fingers to press either the “f-” or “j-” key to indicate whether the probe corresponded to the encoding phase or not. Half of the trials were randomly matched, the other half nonmatched. 50% of the nonmatch trials contained a color that was different from any of the colors from the encoding array; the other 50% displayed a color from a different location in the encoding array. During the task, working memory load was intermixed and randomized resulting in half of the trials being high working memory load and half of the trials low working memory load. High memory load consisted of at least two squares more than for the low memory load. The memory load of each participant was individually titrated at the beginning of the session and participants were categorized as high, average, or low performers. This titration of the task served the purpose of keeping participants engaged by avoiding both floor (accuracy <60%) and ceiling effect (accuracy >90%). The main purpose of the working memory task was to create a uniform brain state to minimize carry-over effect from one breathing condition to the next. In addition, this design enabled us to explore cognitive function immediately after the breathing activity.

#### Breathing tasks

Participants were first demonstrated how to perform three different yoga-inspired breathing techniques, including how to form a particular hand shape (mudra, see Fig 1) to manipulate the nostrils. After ensuring individual mastery of the breathing techniques, participants practiced computer-paced breathing while performing the three introduced breathing techniques sequentially (paced breathing). During the subsequent breathing task iterations, the following three different breathing conditions were investigated in random order within each participant: left nostril breathing (LNB, closing right nostril with thumb); right nostril breathing (RNB, closing left nostril with ring finger and pinkie); and alternate nostril breathing (ANB, creating an anchor with the index and middle finger and closing right and left nostril alternatingly). All participants were instructed to use their right hand (regardless of handedness). We paced the breathing to 6 breaths per minute (0.1Hz) with each breath cycle consisting of 3 seconds inhale, 2 seconds hold, 4 seconds exhale, 1 second hold. To facilitate a steady breathing frequency of 0.1Hz, we showed participants an animated vertical bar that was filling up for the inhales and emptying for the exhales accompanied by a tone of rising and falling pitch for inhales versus exhales, respectively [15]. After reach breathing condition, participants filled in the Positive and Negative Affect Schedule (PANAS) to assess instantaneous mood [16].

### EEG analysis and statistics

#### Preprocessing

EEG data were analyzed offline using scripts developed in MATLAB 2022b (The MathWorks, Inc., MA, USA) together with the EEGLAB toolbox (EEGlab 2022.1 [17]). The data was bandpass filtered from 1Hz to 50Hz and subsequently down sampled to 250Hz. Artifacts were removed via a standard pipeline [18] using the clean_artifacts function from EEGLAB (Channel criterion: 0.8; Line noise criterion: 4; Burst Criterion: 20). Non-brain components (e.g. muscle artifacts and eye-blinks) were excluded from the data by independent component analysis (ICA [19, 20]). Presumed non-neuronal components were removed with the ICLabel function [21]. Specifically, independent components labeled as suspected muscle and eye-movement artefacts were dismissed when assigned probability exceeding 90%. On average, 112.5 ± 7.1 ICA components were retained for the subsequent analyses. From the preprocessed data, we first extracted the breathing signals based on recorded time stamps and separated the EEG data into resting and the different breathing conditions (LNB, RNB, ANB). Spectral analysis was performed on 300 second segments for each breathing condition. Power spectra were calculated based on a frequency range from 1Hz to 30Hz. Similarly, resting state data were obtained for each participant for segments of 180 seconds for eyes-open and 120 seconds eyes-closed.

#### Oscillation power

Individual alpha frequency was determined from the eyes-closed condition, starting at 6Hz as the lower edge. The resulting individual alpha frequency band ranged from -1Hz to +1Hz around the individual peak frequency. Topographic distribution of oscillation power was computed and compared between the resting state and the paced breathing conditions as well as between the three breathing conditions for the individualized alpha and canonical theta (4-8Hz) and beta (12-30Hz) frequency bands. Statistical testing was performed by pairwise t-tests for each electrode location. In addition, for the frequency bands that showed significant differences between breathing conditions and resting state, we performed a region of interest (ROI) exploratory analysis by computing the mean power for a bilateral central-parietal and a frontal midline area. ROIs were refined as electrode set for left central-parietal (36, 37, 41, 42, 52, 53, 47), right central-parietal (86, 92, 98, 93, 87, 104, 103), and frontal midline (4, 5, 9, 10, 12, 15, 16, 18, 19, 22). Control for multiple comparisons was performed with cluster permutation [22].

#### Functional connectivity

To investigate whether there is a difference in functional connectivity between RNB and LNB, we extracted specific electrode labels to pinpoint regions of interest within both the right and left hemispheres of the brain for subsequent examination of functional connectivity. We determined these regions based on the calculated differences in alpha/mu power and conducted statistical analyses using pairwise t-tests. These tests allowed us to define seeds for the functional connectivity analysis using weighted phase lag index (wPLI). This measure of functional connectivity was chosen since it is a commonly used metric of functional connectivity in EEG and exhibits several advantages over other methods [23]. First, by only considering phase and not amplitude it is less likely to be contaminated by fluctuations in amplitude of the signals of interest. Second, by focusing on non-zero phase offsets, wPLI reduces the likelihood of spurious connectivity caused by volume conduction. Third, by using the weighted version of PLI longer phase leads and lags contribute more significantly, which provides further nuance and robustness to noise over the original PLI method. As alpha power differences were bilateral, we designated a right seed (electrodes 86, 87, 92, 93, 98, 103, 104) and a left seed (electrodes 37, 40, 41, 46, 47, 52, 53). For the theta band, we chose a seed in the frontal-midline region (electrodes 4, 5, 9, 10, 12, 15,16, 18, 19, 22). Channels surrounding the seeds were removed from the analysis. Connectivity was averaged over participants. Based on the changes in functional connectivity, we were able to examine the brain network activity patterns between RNB and LNB conditions, helping us discern any potential lateralization effects.

#### Statistical analysis and correlation of non-EEG data

We recorded heart rate (with a 2-lead EKG) and respiratory frequency (with a breathing belt) during the entire session. These data were preprocessed in MATLAB [24]. Heart rate variability was computed as the standard deviation of the interbeat intervals. Statistical analysis was done in R using one-way repeated-measure ANOVA followed by pairwise t-tests as appropriate. Similarly, the working memory data were first preprocessed in MATLAB and then analyzed using R. Due to the limited sample size, we did not investigate sex differences within our data. Additionally, since only two participants were left-handed, no analysis by handedness was performed.

## Results

The primary goal of our study was to investigate if three distinct types of paced nostril breathing–left nostril breathing (LNB), right nostril breathing (RNB), and alternate nostril breathing (ANB)—differentially modulate brain network oscillations measured by EEG. Specifically, we determined differences in spectral power of oscillation frequencies associated with sensorimotor function, the alpha/mu and beta frequency bands in central and parietal areas, and with top-down (cognitive) control, the theta frequency band. Based on the spectral power analysis, we subsequently examined changes in functional connectivity for the frequencies and seed locations with modulation of oscillation power during the breathing task.

### Neuronal oscillations

**Fig 2:**
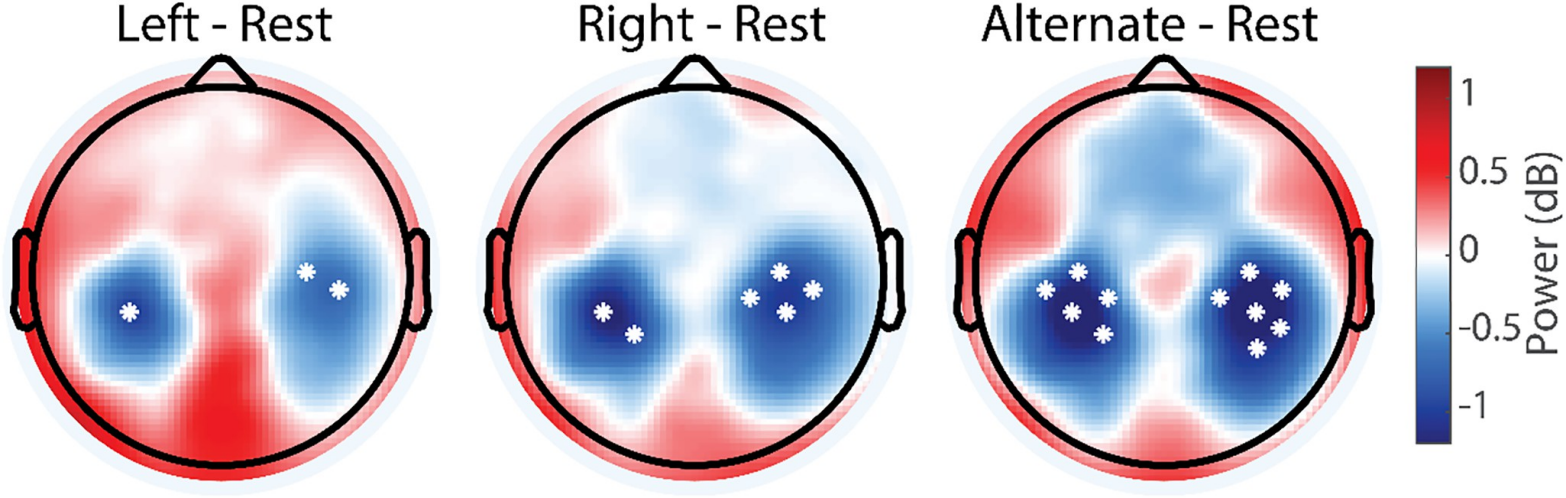
Topographic maps of difference in individual alpha/mu power for left, right, and alternate nostril breathing compared to resting state. In all three nostril breathing conditions, there was a bilateral suppression of power in this alpha/mu frequency when compared to eyes-open resting state. The parietal (post central gyrus) localization suggests this power modulation corresponds to mu oscillations. Asterisks indicate the localized significance of pairwise t-testing.

We first asked if neural oscillations differed between paced nostril breathing and resting state in which the participants were freely breathing and asked to think about “nothing in particular” while keeping their eyes open. We examined an a-priori hypothesized frequency band that encompasses alpha (+/- 1 Hz centered on individual peak) and mu oscillations, which are typically suppressed during sensory processing, movement planning and execution, and task engagement. Each of the three breathing conditions exhibited a symmetric, bilateral suppression of power in this frequency band. The suppression localized to central-parietal locations (Table 2), associated with the mu rhythm [25, 26] (Fig 2).

**Table 2:** Alpha power for left and right central-parietal ROI.

*mean ± std*	*Left Central-Parietal ROI*	*Right Central-Parietal ROI*
**Rest**	0.83 ± 4.66	1.29 ± 4.14
**LNB**	0.23 ±4.86	0.80 ± 4.30
**RNB**	-0.03 ± 4.50	0.45 ± 4.10
**ANB**	-0.22± 4.55	0.18 ± 4.00

Analysis of the average power in the left and right central-parietal areas showed a significant bilateral reduction for ANB and a unilateral right central-parietal reduction for RNB when compared to the resting state (Table 3). However, no significant electrodes survived comparison for multiple comparisons by cluster permutation.

**Table 3:** Difference in alpha power between breathing conditions and resting state for left and right central-parietal ROI.

*ROI*	*Contrast*	*M*	*SD*	*df*	*t*	*p*	*Cohen’s d*
**Left**	**LNB–Rest**	-0.60	2.10	18	-1.25	0.11	-0.12
	**RNB–Rest**	-0.86	2.20	18	-1.72	0.050	-0.18
	**ANB–Rest**	-1.05	2.26	18	-2.02	**0.030**	-0.22
**Right**	**LNB–Rest**	-0.48	1.74	18	-1.20	0.12	-0.11
	**RNB–Rest**	-0.83	1.90	18	-1.90	**0.038**	-0.19
	**ANB–Rest**	-1.10	1.92	18	-2.50	**0.01**	-0.26

**Fig 3:**
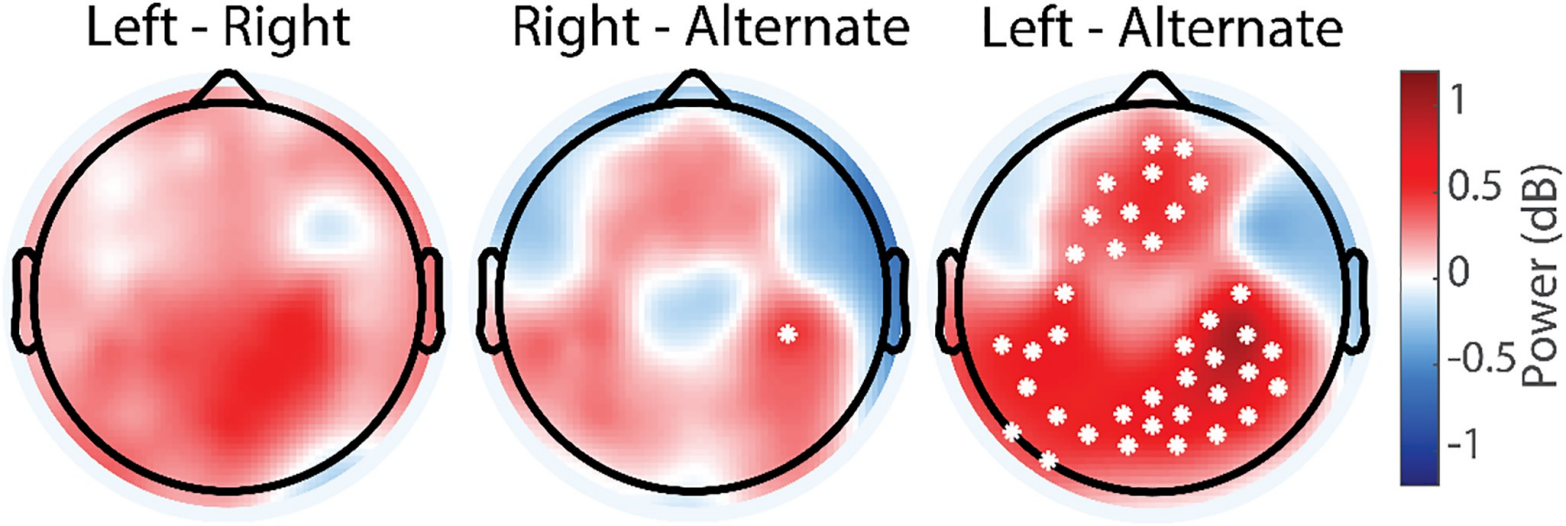
Topographic maps of difference in individual alpha/mu power between breathing conditions. Difference between left and right (left panel), right and alternate (middle panel), and left and alternate nostril breathing (right panel). Alternate nostril breathing significantly differed from left nostril breathing in terms of both parietal bilateral and frontal midline alpha/mu oscillation power. A qualitatively similar pattern occurred for the comparison of right to alternate nostril breathing but did not meet criterion for statistical significance. Note that none of the conditions exhibited an increase in alpha/mu power (see Fig 2) and that the differences here reflect differential levels of suppression of power in this frequency band.

We next examined if this suppression differed between breathing conditions (Fig 3). We found significant differences between the LNB and ANB conditions; the bilateral suppression of the mu rhythm was stronger for ANB when compared to LNB, both at the level of individual electrodes and for the ROI analysis of left and right central-parietal areas (Table 4).

**Table 4:** Difference in alpha power between breathing conditions for left and right central-parietal ROI.

*ROI*	*Contrast*	*M*	*SD*	*df*	*t*	*p*	*Cohen’s d*
Left	LNB–RNB	0.26	1.25	18	0.90	0.20	0.052
	RNB–ANB	0.19	1.00	18	0.83	0.21	0.040
	LNB–ANB	0.45	1.05	18	1.86	**0.040**	0.090
Right	LNB–RNB	0.35	1.17	18	1.30	0.11	0.080
	RNB–ANB	0.27	0.93	18	1.29	0.11	0.065
	LNB–ANB	0.62	0.98	18	2.77	**0.0063**	0.14

The topographic differences survived controlling for multiple comparisons with cluster permutation. For the comparison of RNB with ANB, the topographical distribution looked similar but of lower magnitude, though this difference was not statistically significant. In addition, we found a difference in frontal midline alpha power, which reached statistical significance for the comparison of LNB with ANB only. Finally, there was no significant difference between the left and right unilateral breathing conditions and there was no lateralization of mu oscillation power when computed as the difference between left and right hemispheric power in the region of interest based on the localization of the mu suppression (t = -1.00, N = 19, p = 0.33).

**Fig 4:**
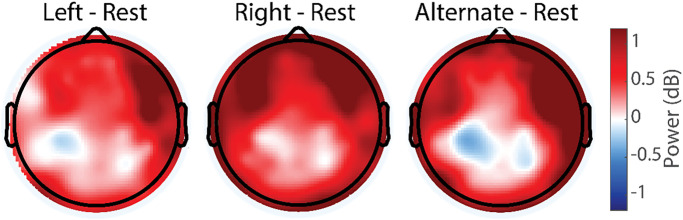
Topographic maps of difference in beta power. Topographic maps of difference in beta power for left, right, and alternate nostril breathing. In all three nostril breathing conditions, there was no significant modulation of beta oscillation power compared to eyes-open resting state.

Beta oscillations play a significant role in the motor system. Given our findings of the effect of breathing on the mu rhythm associated with somatosensory processing and potentially the motor component of holding a nostril closed while performing visually paced breathing, we next examined if the three nostril breathing conditions also impacted beta oscillations when compared to rest. We found no significant difference in beta oscillations when comparing to the resting-state condition for all three nostril breathing conditions (Fig 4).

Finally, given the role of frontal-midline theta oscillations in top-down control and thus potentially the regulation of breathing, we also determined the topographic distribution of the change in theta oscillation power when comparing the breathing conditions to the resting state. We found that paced nostril breathing increased frontal theta oscillations at the midline (Table 5).

**Table 5:** Theta power for frontal midline ROI.

*Condition*	*Theta power (mean ± std)*
**Rest**	0.017 ± 1.96
**LNB**	0.64 ± 2.60
**RNB**	0.66 ± 2.60
**ANB**	0.99 ± 2.44

**Fig 5:**
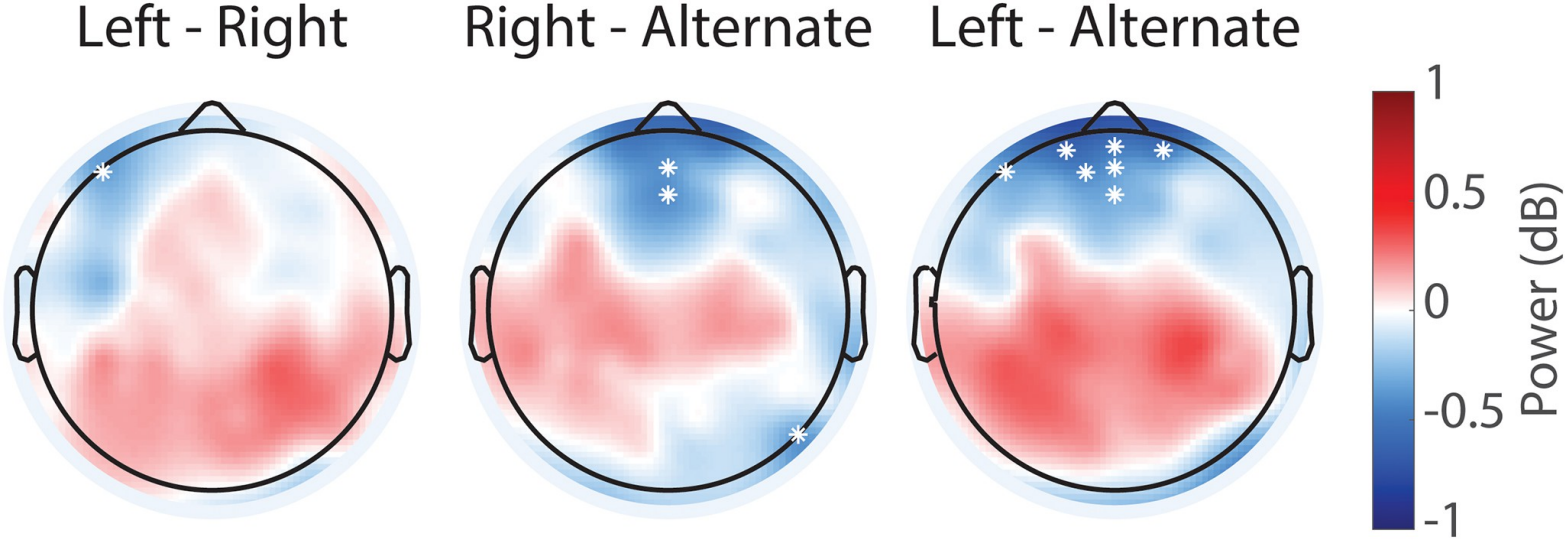
Topographic maps of difference in theta power for left, right, and alternate nostril breathing compared to resting state. Topographic maps of difference in theta power for left, right, and alternate nostril breathing compared to rest. In all three nostril breathing conditions, there was a significant increase in theta oscillation power in the frontal midline and occipital areas.

Like the findings in the mu frequency band, the increase in frontal midline theta was more pronounced for the ANB condition (Fig 5). The cluster of significant electrodes survived controlling for multiple comparisons. Comparison of the theta power for the frontal midline ROI also showed significant increase in theta oscillations for all three breathing conditions when compared to the resting state (Table 6).

**Table 6:** Difference in theta power between breathing conditions and resting state for frontal midline ROI.

	*M*	*SD*	*df*	*T*	*p*	*Cohen’s d*
**LNB–Rest**	0.62	0.91	18	2.98	**0.0040**	0.26
**RNB–Rest**	0.64	1.14	18	2.44	**0.013**	0.27
**ANB–Rest**	0.98	0.91	18	4.68	**<0.0001**	0.42

**Fig 6:**
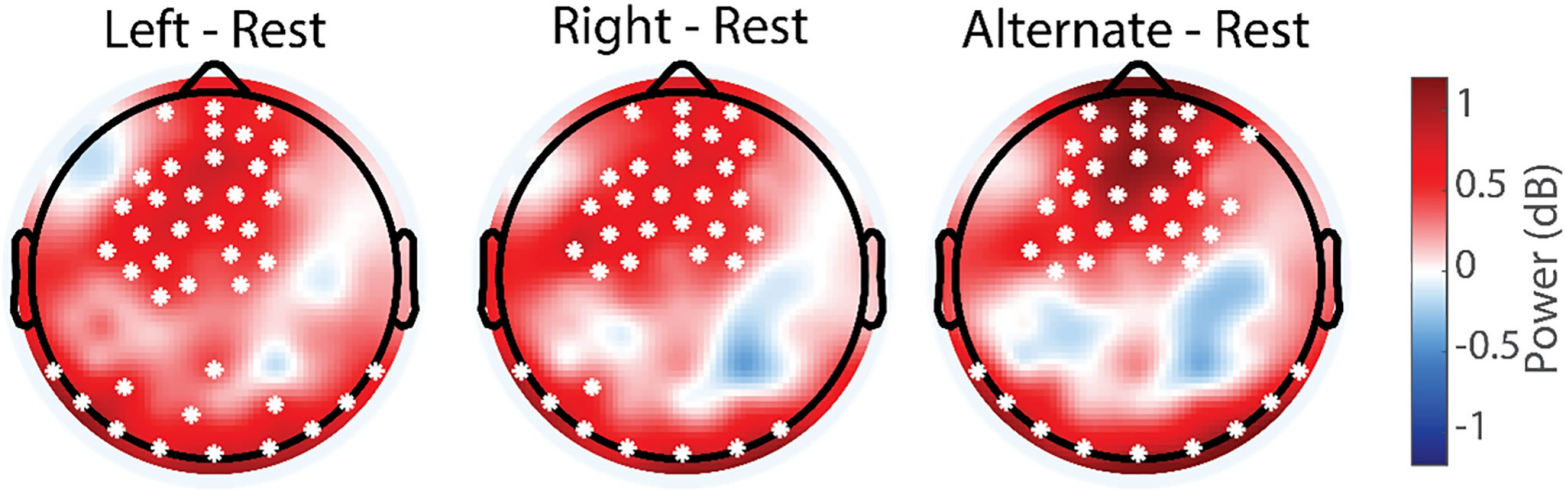
Topographic maps of difference in theta power between breathing conditions. Difference between left and right (left panel), right and alternate (middle panel), and left and alternate nostril breathing (right panel). Alternate nostril breathing was associated with increased frontal midline theta power.

The direct comparison of the three breathing conditions showed only a few electrodes with a significant reduction in theta oscillations (Fig 6), which did not survive controlling for multiple comparisons. Mean power in the midline frontal ROI was significantly elevated for ANB when compared to either RNB or LNB (Table 7).

**Table 7:** Difference in theta power between breathing conditions for frontal midline ROI.

	*M*	*SD*	*df*	*t*	*p*	*Cohen’s d*
**LNB–RNB**	-0.018	0.57	18	-0.14	0.45	-0.0068
**RNB–ANB**	-0.34	0.95	18	-1.54	0.070	-0.13
**LNB–ANB**	-0.35	0.77	18	-2.01	**0.030**	-0.14

Together, our EEG findings of changes in oscillation power demonstrate that the organization of neural oscillations during paced nostril breathing differs from resting state in the alpha/mu and theta frequency bands. Overall, alternate nostril breathing was associated with more pronounced modulation of neuronal oscillations compared with unilateral nostril breathing. Given the role of oscillations in coordinating large-scale networks, we next examined if functional connectivity differed between the breathing conditions.

### Functional connectivity

We focused our functional connectivity analysis on the alpha/mu and theta frequency bands that showed power modulation with the breathing task. We examined connectivity from the regions with greatest oscillation power modulation, resulting in regions of interest localized to left and right central-parietal cortex for alpha/mu and frontal midline for theta oscillations, respectively. The analysis compared LNB and RNB to exclude potential confounds from the hand movement required for ANB.

**Fig 7:**
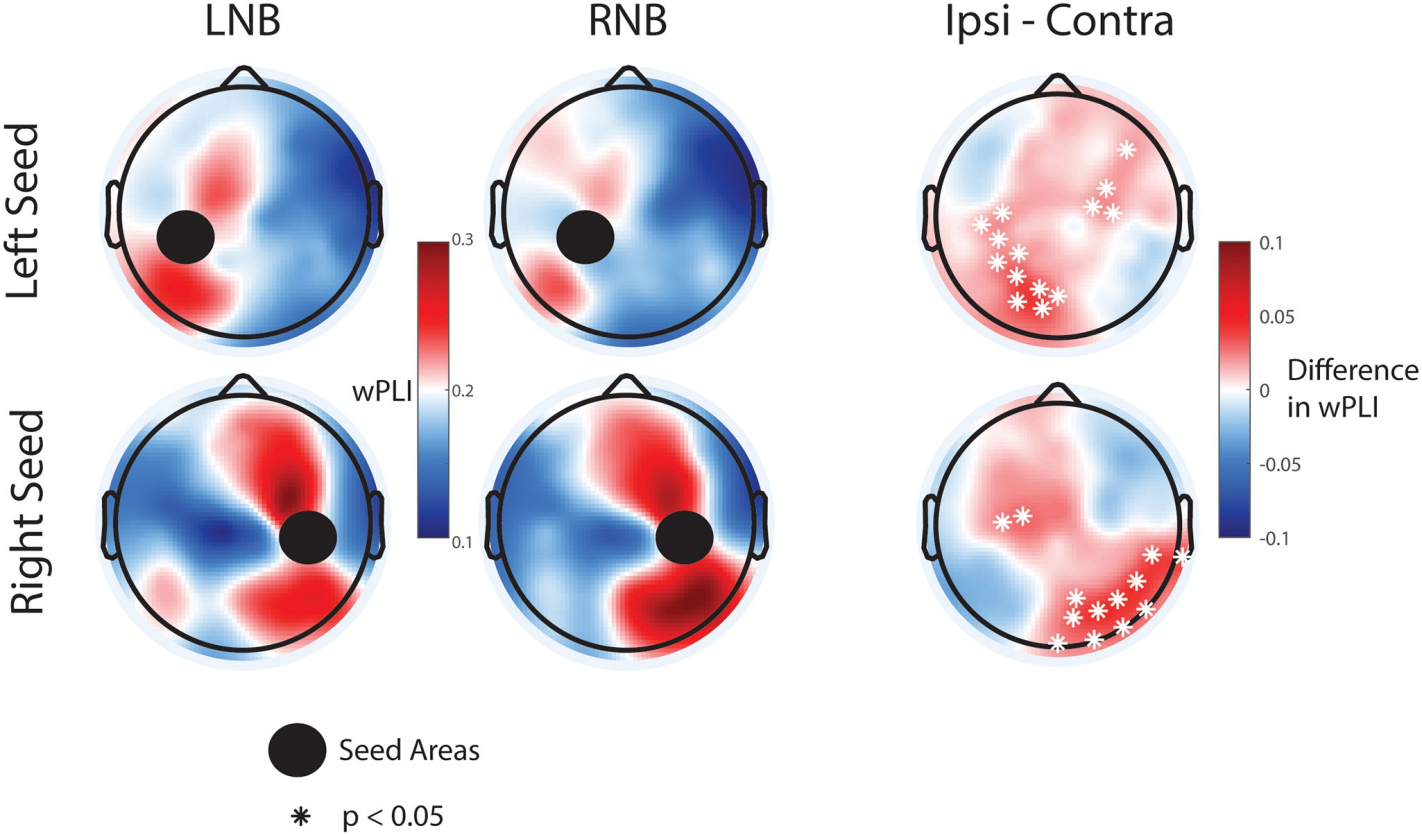
Topographic maps of difference in alpha functional connectivity between breathing conditions. Top: Alpha functional connectivity for left central-parietal seed (86, 87, 92, 93, 98, 103, 104). LNB, RNB, and difference for ipsi- versus contralateral breathing conditions. Bottom. Alpha functional connectivity for right central-parietal seed (86, 92, 98, 93, 87, 104, 103). LNB, RNB, and difference for ipsi- versus contralateral breathing conditions.

For the alpha/mu band, we hypothesized connectivity to be higher for the ipsilateral than the contralateral hemisphere due to the inhibitory role of this frequency band and the contralateral innervation of cortex. In agreement with this hypothesis, we found significantly higher levels of connectivity in the left posterior hemisphere for the left central-parietal ROI for LNB versus RNB (Fig 7). Similarly, for right central-parietal ROI connectivity was higher for the RNB versus LNB (Fig 7). Since neither the LNB nor RNB condition included any hand movement, the most parsimonious explanation for these findings is a differential activation of hemispheric inhibitory connectivity ipsilateral to the nostril pathway, likely reflecting the somatosensory input caused by air movement.

**Fig 8:**
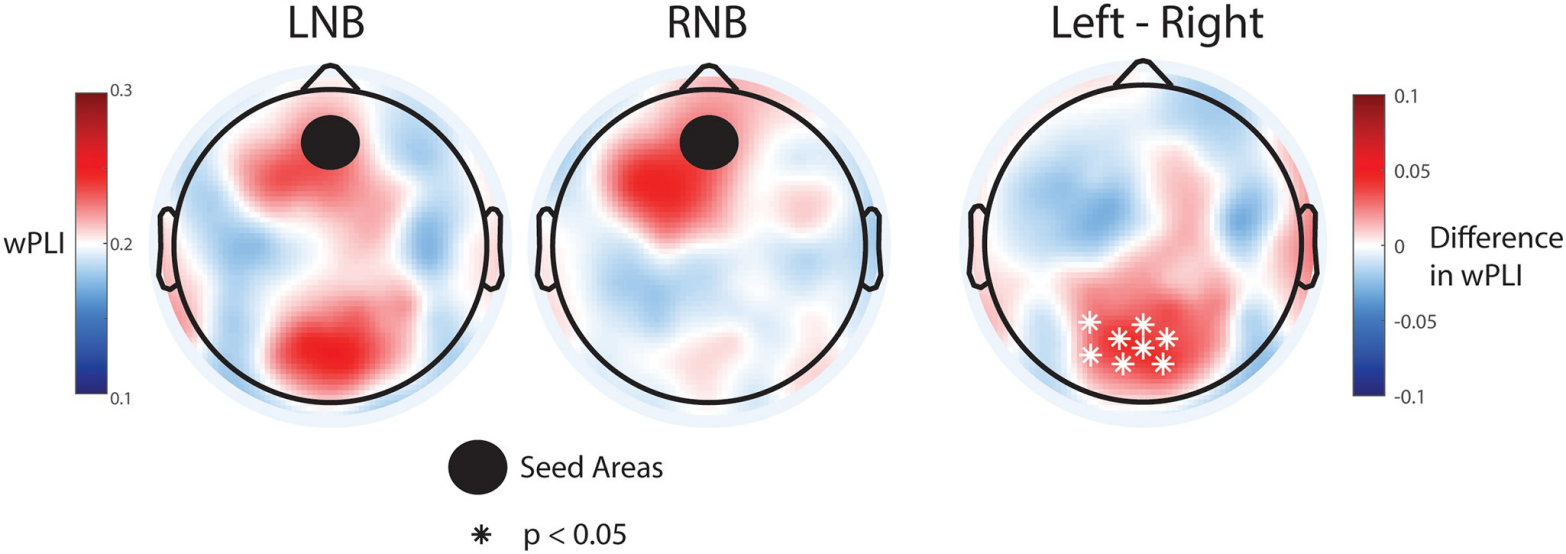
Topographic maps of difference in theta functional connectivity between breathing conditions. LNB (left), RNB (middle), LNB–RNB (right). Seed area: frontal midline (4, 5, 9, 10, 12, 15,16, 18, 19, 22).

For the theta functional connectivity with the midline frontal location as the ROI, we found a significant difference in connectivity to the posterior midline. Specifically, LNB was associated with a marked anterior-posterior midline connectivity, which was absent for RNB (Fig 8).

### Cognitive function and mood differences

**Fig 9:**
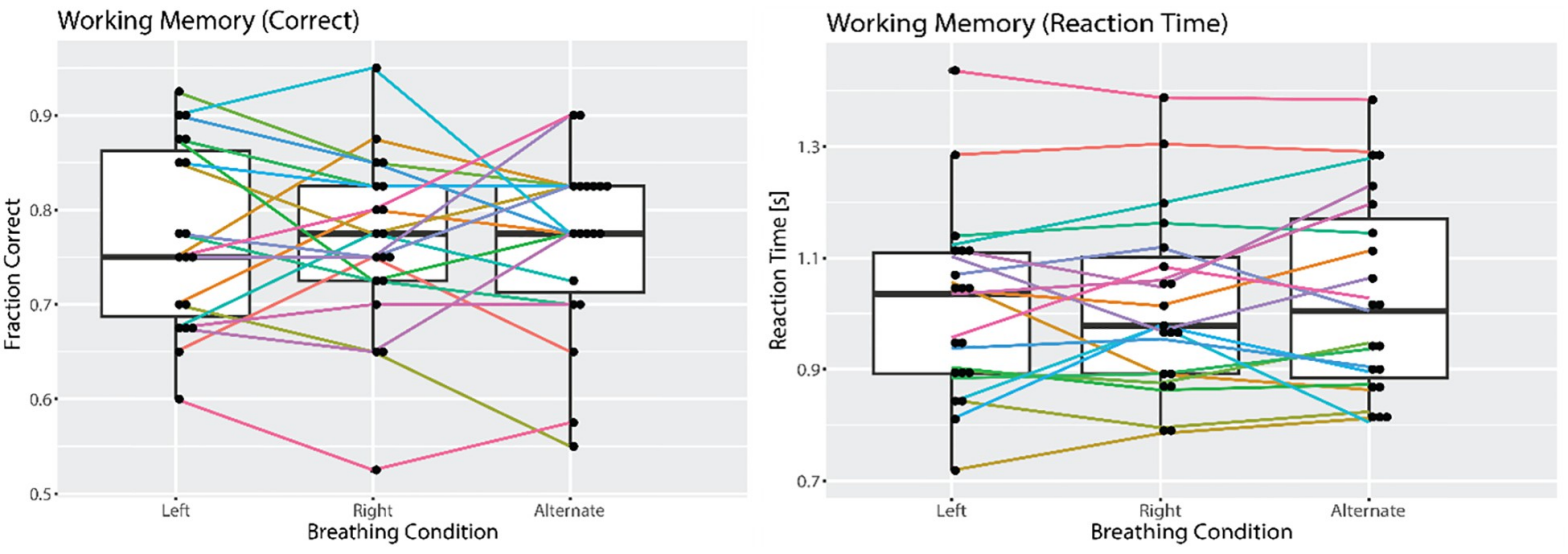
Working memory performance. There was no difference in working memory performance measured as fraction of correct trials (left) and reaction time for correct trials (right).

Participants performed a visuospatial working memory task in-between breathing conditions primarily to limit any carry-over effect. As a secondary outcome, we analyzed the relative impact of breathing conditions on working memory performance. One-way ANOVA did not support an impact of the breathing conditions on reaction time (F(2,36) = 0.539 p = 0.59 N = 19) or number of correct answers (F(2,36) = 0.065 p = 0.94 N = 19) (Fig 9).

**Fig 10:**
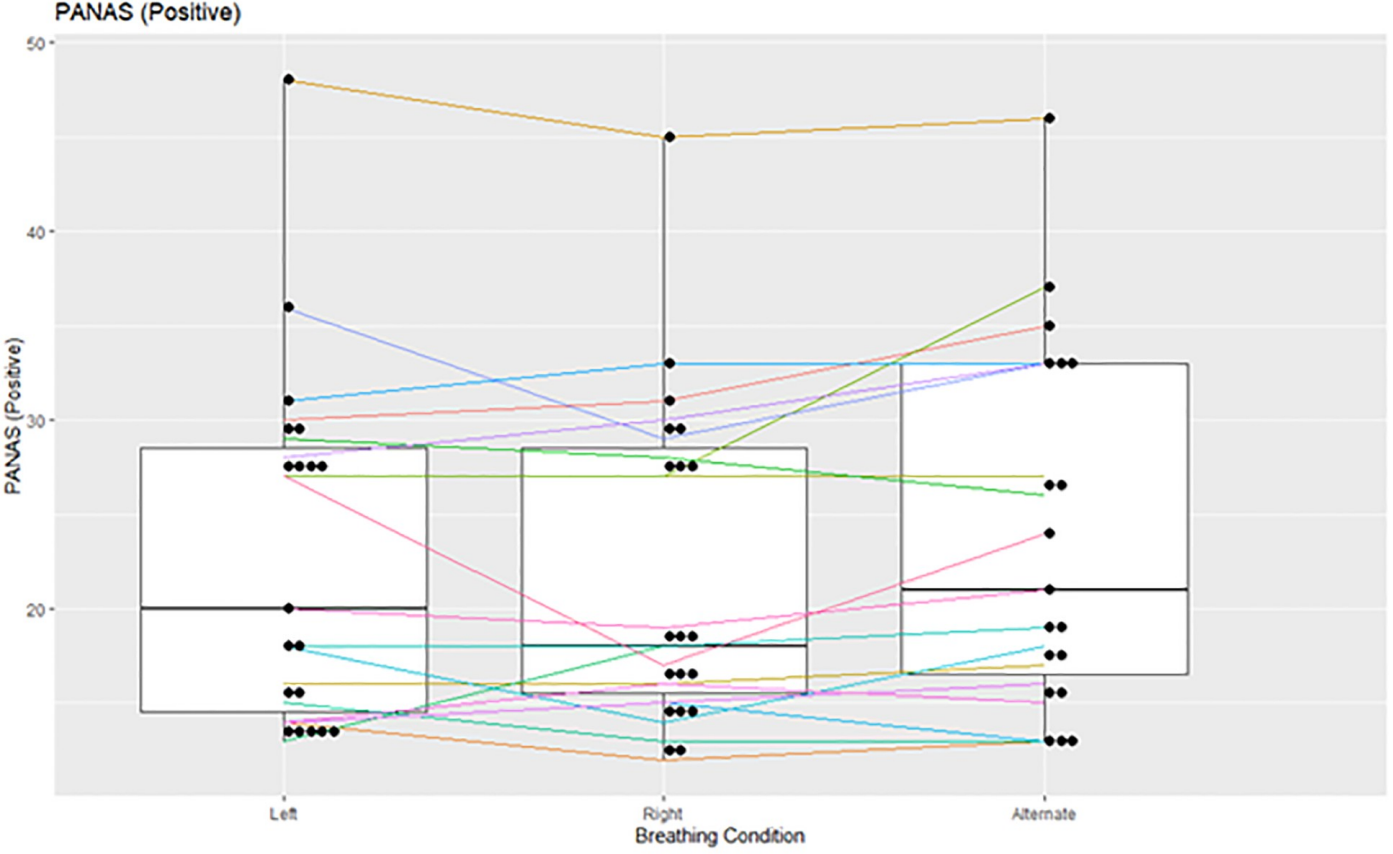
PANAS positive. Alternate Nostril Breathing (ANB) slightly increased positive mood when compared to Right Nostril Breathing (RNB).

After each breathing task, participants were asked to complete a PANAS questionnaire to examine if there was a connection between any breathing condition and mood. We only found a trend-level overall effect of breathing condition on positive mood, with ANB associated with slightly increased positive mood when compared to RNB (F(2,36) = 2.97 p = 0.064 N = 19; ANB–RNB: p = 0.015) (Fig 10). There was no effect of breathing condition on negative mood (F(2,36) = 1.099 p = 0.344 N = 19).

### Non-neuronal physiology

**Fig 11:**
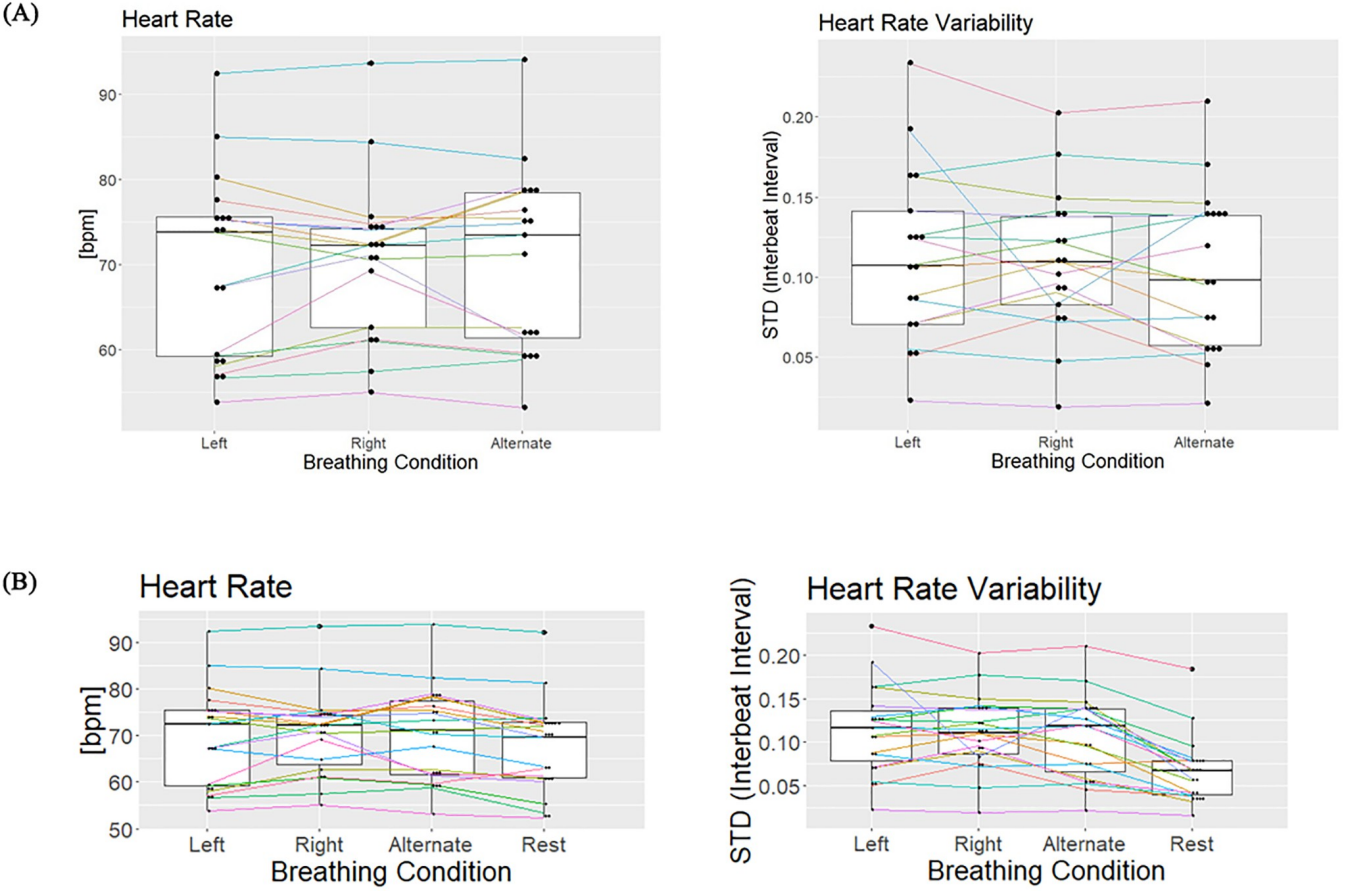
**A.** There was no difference in heart rate measured in bpm (left) and heart rate variability measured by the standard deviation of the interbeat intervals (right) between breathing conditions. **B.** ANB and RNB significantly increased heart rate compared to resting state. Generally, paced nostril breathing significantly increased HRV compared to rest.

During the entire session, we recorded the heart rate with two ECG electrodes. We found no significant effect of the breathing technique on heart rate (F(2,32) = 0.495 p = 0.614 N = 17), indicating that each breathing condition (RNB, LNB, and ANB) had similar effects on heart rate (Fig 11A). When comparing each breathing technique to resting state, however, we found statistical significance for ANB (p = 0.004) and RNB (p = 0.008), but not for LNB (p = 0.1) (Fig 11B). Similarly, there was no significant difference in heart rate variability between breathing conditions (F(2,34) = 1.046 p = 0.363 N = 17) (Fig 11A) but breathing significantly increased HRV compared to rest (Fig 11B). The respiratory rate across participants during the breathing conditions was consistently at 0.1Hz showing proper entrainment and pacing.

## Discussion

Our results suggest widespread cortical activation during paced nostril breathing with reductions in inhibitory alpha/mu rhythms when compared to eyes-open resting state in participants naïve to yoga breathing techniques. We found a bilateral decrease in central-parietal alpha/mu power and an increase in frontal midline theta power with the largest effect recorded for ANB. In contrast, beta oscillations were not significantly affected by the paced nostril breathing when compared to resting state. A functional connectivity analysis showed a higher ipsilateral connectivity in the alpha/mu frequency band and an anterior-posterior midline theta band connectivity, for LNB but not RNB.

The decrease of oscillation power in the alpha/mu frequency band was localized to the central-parietal regions corresponding to somatosensory cortex, which is more anterior than the usual occipital localization of resting-state alpha oscillations. This localization suggests a suppression of the mu rhythm rather than the occipital alpha rhythm associated with visual processing and attention. The mu rhythm usually occupies the same frequency band as alpha (8-13Hz) and it is considered to be part of the mirror neuron system [25]. Mu suppression occurs during sensory processing involved in voluntary movement [27]. Moving or even thinking about moving the hand or a finger will attenuate the mu rhythm in the contralateral hemisphere [26]. Mu suppression tends to be more prominent when learning a new movement task [27]. While little is known about the mu rhythm in paced nostril breathing tasks, our findings are in general agreement with these features of the mu rhythm. We found the most prominent and widespread mu rhythm suppression during ANB, which was the most challenging condition in terms of sensorimotor processing since participants had to make periodic small hand movements to block the right or left nostril in an alternating way for each breath. Our study did not directly assess the effect of the pacing for the three breathing conditions when compared to rest. Slow paced breathing at a rate of 0.1Hz (compared to faster paced breathing) without hand manipulation has been shown to increase power in both the alpha and theta frequency bands [28]. This contrasts with our findings of opposite modulation of these two bands when compared to rest. Potential explanations will require future experimental investigation. Overall, our results of opposite modulation of theta and alpha oscillations point towards increased cortical activation as theta oscillations index arousal [29] and overall cognitive effort [13].

We did not find significant changes in the beta frequency range. Beta oscillations reflect inhibitory activity in the motor system and are suppressed at the initiation of motor action. The lack of modulation of beta oscillations suggests that the cortical activation indexed by the mu suppression may be at least partially the result of mirroring and following the breathing instructions. The lack of modulation of beta oscillations does not per se preclude a contribution by a confound related to the more complex hand movement required for ANB. Accordingly, a study on uninostril breathing, which involved delivering air pressure through a device, found changes in the beta frequency band that were widely distributed [8].

Research indicates that conditions like hypocapnia and loaded breathing during wakefulness can give rise to preinspiratory potentials [30, 31]. However, these respiratory-related premotor potentials predominantly manifest as involuntary responses to respiratory discomfort induced by moderate inspiratory or expiratory loads [32]. In our study, participants voluntarily manipulated nasal airflow without any imposed or forced breathing constraints. While nostril dominance may have influenced air resistance–given that breathing through the non-dominant nostril has been shown to impact posterior brain areas [8], our data revealed a more significant suppression of the mu rhythm during Alternate Nostril Breathing (ANB) compared to Unilateral Nostril Breathing (UNB). This finding aligns with previous research indicating that changes in mu rhythm are primarily driven by motor activity [33], suggesting that the observed effects on cortical oscillations are largely influenced by factors beyond airflow.

Our data also show an increase in the oscillation power in the theta frequency band which occurred most prominently in the frontal midline region of the brain. This localization of the frequency change is in line with existing literature on frontal midline theta power in focused attention and self-regulation [34–37]. One study looked at focused attention and breath control in Zen practitioners and found increased frontal midline theta as a function of mental concentration and attributed this process to a regulatory effect of the frontal neural network over the autonomic nervous system [36]. Another study suggested an implication of the anterior cingulate cortex and the prefrontal cortex during self-regulation and cognition and found correlations with parasympathetic activity during meditative processes [37]. Brandmeyer et al. suggest an inverse correlation between increased theta as a measure of top-down control and increased alpha during mind wandering and rumination [35]. While none of these studies looked at paced nostril breathing, they support our findings of increased frontal midline theta oscillation power during the focused breathing tasks. Participants had to concentrate to align their inhales and exhales with the pacing stimuli that were presented to them, and, for ANB, additionally coordinate their hand movements. Indeed, we see the most prominent change in theta oscillation power during the ANB condition. We also found that ANB most significantly increased participants’ heart rate when compared to resting state–probably because more cognitive effort was required to coordinate breathing and movement. Furthermore, we found that paced nostril breathing increased heart rate variability compared to resting state, which suggests a potential shift towards parasympathetic activity. This is in line with previous studies on resonance breathing and cardiac coherence [38–41]. Specifically, breathing at 0.1Hz has been demonstrated to mediate a strong synchronization of cardiac and cortical activities [38]. In line with this literature, we propose that the pacing of the nostril breathing influences autonomic function.

Two previous studies using EEG supported the hypothesis that nostril breathing affects brain activity by linking the nasal cycle to alternating cerebral hemispheric activity [11, 12]. One study found that nasal airflow correlated with a contralateral increase in brain activity. The authors showed that predominant airflow through one nostril was associated with a broadband increase in oscillation power measured by EEG in the contralateral hemisphere [11]. Similarly, manually blocking one nostril and intentionally breathing through the other has been shown to increase the broadband EEG signal in the contralateral hemisphere [12]. Other studies, however, failed to demonstrate an asymmetry in EEG power associated with alternate nostril breathing [9, 10]. Instead, there was a relative reduction in theta power during alternate nostril breathing, followed by a lower amplitude of beta oscillations after alternate nostril breathing [10]. The small number of electrodes in this study, however, do not allow for spatial localization of these changes in oscillation power. Recently, a study found a general decrease in EEG spectral power during uninostril breathing compared to a resting-state baseline with the most changes occurring in the beta frequency band [8]. Additionally, when compared to right nostril breathing, left nostril breathing was shown to reduce spectral power in the delta, theta, and alpha band except in the posterior areas of the brain where there was no reduction or difference. Overall, the literature on EEG modulation by breathing techniques is sparse and suffers from small sample size and limited electrode coverage due to low electrode counts [10, 42, 43]. Commonly, previous studies examined the alpha oscillation as a marker of “relaxation” or “meditative state.” [44] Using high-density EEG, we demonstrated that the change in this frequency power is localized and lateralized. Previous studies typically computed and discussed the change in oscillation across canonical frequency bands and often reported effects of similar directionality (e.g., suppression or enhancement) across these bands [11, 12]. Given that different oscillations correspond to excitatory and inhibitory processes, such findings are difficult to interpret. For example, a concomitant increase in theta oscillations (increased effort and engagement) appears contradictory to an increase in inhibitory alpha or beta oscillations. Our results are internally consistent in terms of their support for an overall increase in cortical excitability when compared to rest and when comparing alternate to unilateral nostril breathing.

Functional connectivity analysis provides an additional level of understanding of cortical oscillations. Long-range synchronization of neuronal rhythms represents a key mechanism for communication between brain areas [45]. Intriguingly, we observed lateralization by nostril for functional connectivity but not for oscillation power. Consistent with the general finding that the ipsilateral cortex displays higher levels of inhibitory oscillations than the contralateral cortex for sensory input, alpha/mu functional connectivity was greater ipsilateral to the nasal pathway used. Connectivity in this frequency band has not been previously explored and will require replication in future studies. In the theta frequency band, we noticed anterior-posterior midline connectivity between left and right nostril pathways. The source of this difference remains unclear; however, it aligns conceptually with pranayama beliefs, which suggest that left nostril breathing is relaxing [5]. Similarly, high trait anxiety is associated with reduced midline theta frontal-parietal connectivity [46]. Furthermore, positive emotional experience during meditation correlates with an increase in frontal-parietal theta oscillations [34]. Thus, the increase in midline frontal-parietal theta connectivity for the left nostril pathway may represent the mechanism of the suggested relaxing effect of this specific breathing technique.

Finally, on a more technical note, the functional connectivity findings revealed distinct differences between the left and the right nostril pathways. These differences are unlikely due to be confounded by hand movement as participants were instructed to keep their hand still while closing the contralateral nostril. Additionally, since the pacing was consistent across all three conditions in this study, the left versus right findings cannot be attributed to variations in pacing. The functional connectivity results provide valuable insights into the neural mechanisms underlying nostril breathing and represent a promising area for further research. To our knowledge, only one other study has investigated EEG-based functional connectivity related to nostril breathing [47]. This study used a less tight scientific contrast by comparing nose to mouth breathing and found differences in connectivity for the delta, theta, and beta bands. Anterior-posterior theta connectivity was increased for nose versus mouth breathing, with most of the strengthened connections limited to the left hemisphere. These findings parallel our results that also showed an increase in anterior-posterior theta-frequency connectivity, specifically for LNB. This midline theta connectivity found in both studies may reflect elevated self-referential, default mode network activity, and provide the basis for an overall network marker of breathing techniques associated with modulation of mood and perceptual state. Together, these findings highlight the potential key role of theta functional connectivity in nostril breathing.

As with any scientific study, the results presented here should be interpreted in the light of the limitations of the chosen study design. First, our research involved a relatively small number of participants. However, this limitation is somewhat mitigated by the within participant design, which enhances statistical power, and by the clarity and biological plausibility of the EEG findings regarding of spatial localization, modulation of specific frequency bands, and functional connectivity. Nonetheless, we acknowledge that larger follow-up studies are warranted based on these preliminary results. Second, our study design does not fully isolate hand movement from the resulting changes in nostril airflow. One prior study used a device to control airflow without participant hand manipulation [8] and found predominantly changes in beta oscillation power compared to baseline. Future investigations should also include a control condition that isolates hand movement without blocking nostril pathways. We chose to incorporate traditional yogic hand movements to enhance external validity and to examine the effect of the actual breathing techniques on EEG oscillations. Third, we did not assess nostril dominance at the time of the study visit, limiting our ability to draw conclusions about the effects of these breathing techniques on nostril dominance or change in nasal cycle. Fourth, we cannot determine any potential effects of handedness due to the small number of left-handed participants in our sample. Finally, not all electrode sets identified as statistically significant survived adjustments for multiple comparisons. A larger sample size may help address this issue, particularly given the relatively small effect sizes observed in this study.

Despite these limitations, our study clearly demonstrates modulation of brain network oscillations in association with nostril breathing condition. These findings lay the groundwork for future research into the effects of repeated practice of these breathing techniques on brain activity. Additionally, it may be worthwhile exploring how long these changes in brain activity persist and whether they offer potential therapeutic benefits, particularly for neurological and psychiatric conditions linked to altered autonomic regulation. Given the known advantages of slow, paced breathing, further research on paced nostril breathing could be especially relevant for anxiety disorders.
